# Stable low-level expression of p21^WAF1/CIP1 ^in A549 human bronchogenic carcinoma cell line-derived clones down-regulates E2F1 mRNA and restores cell proliferation control

**DOI:** 10.1186/1476-4598-5-1

**Published:** 2006-01-10

**Authors:** Timothy G Graves, Michael W Harr, Erin L Crawford, James C Willey

**Affiliations:** 1 Departments of Medicine and Pathology, Medical University of Ohio, 219 Health Education Building, 3055 Arlington Avenue, Toledo, OH, 43614-5806, USA

## Abstract

**Background:**

Deregulated cell cycle progression and loss of proliferation control are key properties of malignant cells. In previous studies, an interactive transcript abundance index (ITAI) comprising three cell cycle control genes, [MYC × E2F1]/p21 accurately distinguished normal from malignant bronchial epithelial cells (BEC), using a cut-off threshold of 7,000. This cut-off is represented by a line with a slope of 7,000 on a bivariate plot of p21 versus [MYC × E2F1], with malignant BEC above the line and normal BEC below the line. This study was an effort to better quantify, at the transcript abundance level, the difference between normal and malignant BEC. The hypothesis was tested that experimental elevation of p21 in a malignant BEC line would decrease the value of the [MYC × E2F1]/p21 ITAI to a level below this line, resulting in loss of immortality and limited cell population doubling capacity. In order to test the hypothesis, a p21 expression vector was transfected into the A549 human bronchogenic carcinoma cell line, which has low constitutive p21 TA expression relative to normal BEC.

**Results:**

Following transfection of p21, four A549/p21 clones with stable two-fold up-regulated p21 expression were isolated and expanded. For each clone, the increase in p21 transcript abundance (TA) was associated with increased total p21 protein level, more than 5-fold reduction in E2F1 TA, and 10-fold reduction in the [MYC × E2F1]/p21 ITAI to a value below the cut-off threshold. These changes in regulation of cell cycle control genes were associated with restoration of cell proliferation control. Specifically, each transfectant was capable of only 15 population doublings compared with unlimited population doublings for parental A549. This change was associated with an approximate 2-fold increase in population doubling time to 38.4 hours (from 22.3 hrs), resumption of contact-inhibition, and reduced dividing cell fraction as measured by flow cytometric DNA analysis.

**Conclusion:**

These results, likely due to increased p21-mediated down-regulation of E2F1 TA at the G1/S phase transition, are consistent with our hypothesis. Specifically, they provide experimental confirmation that a line with slope of 7,000 on the p21 versus [MYC × E2F1] bivariate plot quantifies the difference between normal and malignant BEC at the level of transcript abundance.

## Background

Bronchogenic carcinoma (BC) is the leading cause of cancer-related death in the United States and most industrialized nations [1]. It is reasonable to expect that improved mechanistic understanding of BC will lead to reduced death rate through more effective prevention and treatment regimens.

In an effort to identify biomarkers that diagnose BC with more accuracy than cytomorphologic criteria, this laboratory developed and has employed a highly quantitative, quality controlled RT-PCR method, Standardized RT (StaRT)-PCR [2-4]. With StaRT-PCR, data from all experiments may be directly compared in the same database. This leads to synergistically increasing value of data over time as data accumulate. Also, because the data are all numerical and standardized, they are easily combined into interactive transcript abundance indices (ITAI). ITAI typically are more closely associated with the phenotype of interest and are more likely to yield clinically useful biomarkers than values for individual genes, either alone or in multivariate analysis [5-7]. Initially, StaRT-PCR was used to measure the transcript abundance (TA) values of fifteen cell proliferation control genes (including MYC, E2F1, p21, RB1, PCNA, cyclin D2, cyclin D3, and p53) [6]. Although TA values for any single gene did not accurately distinguish normal from malignant human bronchial epithelial cells (BEC), an ITAI in the form of [MYC × E2F1]/p21 (using a cut-off value of 7,000) was accurate [2,6,8].

In previous studies of the association between the [MYC × E2F1]/p21 ITAI and malignancy, it was observed that there is substantial variation in transcriptional regulation of p21 among BC tissues [9]. In some BC samples, p21 expression is low relative to normal BEC, and in others it is markedly elevated. For example, in some BC there is decreased p53 function mediated by a mutation in the p53 regulatory pathway (e.g. p14ARF deletion in A549) [10], resulting in inappropriately low p21 expression and an ITAI above 7,000. In this context, even relatively low levels of MYC and E2F1 are sufficient to cause loss of proliferation control. However, even in BC tissues with high p21, the [MYC × E2F1]/p21 ITAI value is nearly always above 7,000. This suggests that E2F1 and/or MYC are increased to high enough levels and compensatory pathways, represented by p21 TA level, are inadequate to ensure cell proliferation control.

The p21^WAF1/CIP1 ^gene is a key regulator of cell cycling [9]. Up-regulation of p21^WAF1/CIP1^, which occurs mainly at the transcriptional level [11,12], is mediated primarily by the tumor suppressor p53 after genotoxic stress [13]. In turn, p21 protein interacts with cyclin-cdk complexes [14-16], associates with pRb protein [17], PCNA [18-20], GADD45 [21,22], cooperates with 14-3-3 [23], and/or modulates the activities of DNA polymerase δ [24]. These molecular changes are associated with growth arrest [13], senescence, terminal differentiation, apoptosis, and contact inhibition [11]. These and other changes mediated by either p53-dependent [13] and/or p53-independent means (i.e. Sp1/Sp3, Smads, Ap2, STAT, and BRCA1 (reviewed in [25])) contribute to G1 [26] and G2 checkpoint control [27] and regulation of the cell cycle. Experimental up-regulation through ectopic p21 expression [22-26] or stimulation of endogenous p21 transcription [28-30] results in controlled cell proliferation.

The purpose of these studies was to better quantify, as measured by TA values, the relationship among key genes associated with cell proliferation control. Based on previous studies, a cut-off threshold of 7,000 for the [MYC × E2F1]/p21 ITAI accurately distinguishes normal from malignant lung samples. The hypothesis was tested that this empirically derived cut-off is mechanistically determined and is a mathematical representation of the difference between normal BEC and malignant BEC. First, the malignancy ITAI genes MYC, E2F1, and p21 were measured in an additional set of primary normal and malignant lung samples to more firmly establish the threshold separating them. Then, experimental studies were conducted on the human BC line, A549. This line was chosen because it has down-regulated p21 expression, in part due to p14ARF deletions [10], resulting in an ITAI above the cut-off threshold of 7,000. If the hypothesis was correct, stable exogenous expression of p21 in A549 would decrease the ITAI ratio to a level that is below this cut-off. Four A549 clones (1, 29, 30, and 32) that express increased constitutive levels of endogenous and exogenous p21 were established. The mathematical relationship among TA values of the genes comprising the ITAI was measured and the effects on cell cycling and proliferation control were examined.

## Results

### Determination of the ITAI cut-off value that separates normal from malignant BEC

**Figure 1:**
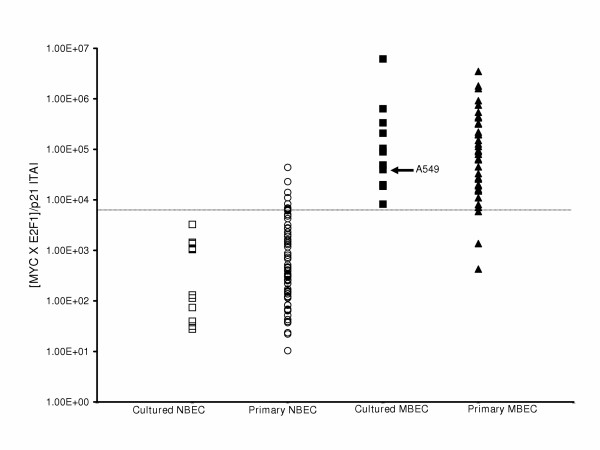
**Histogram analysis of the [MYC × E2F1]/p21 ITAI**. Each data set comprises either cultured or primary normal bronchial epithelial cell (NBEC) or cultured or primary malignant bronchial epithelial cell (MBEC) samples assessed for the ITAI. The ITAI cut-off is indicated by the horizontal line at 7,000 molecules. Parental A549 is indicated.

Previously, we reported that a cut-off threshold of 7,000 for the [MYC × E2F1]/p21 ITAI accurately distinguishes normal from malignant BEC [8]. An additional 65 normal and 31 malignant samples were assessed for this study and the results, combined with data from previous studies, are presented in Figure 1 and in Additional File 1. In these studies, sensitivity and specificity are greater than 90% for distinguishing normal from malignant BEC using a value of 7,000 for the ITAI. Among primary samples, there are numerous factors not present in cell lines that may contribute to false negatives and/or false positives. False positives (high index value in morphologically non-malignant samples) may be due to poor sample quality and RNA degradation or incorrect morphological diagnosis. False negatives (low index value in morphologically malignant samples) may be due to small fraction of tumor cells in the biopsy sample, or incorrect morphological diagnosis.

### Describing the ITAI threshold as a linear equation derived from cell cycle gene TA values

**Figure 2:**
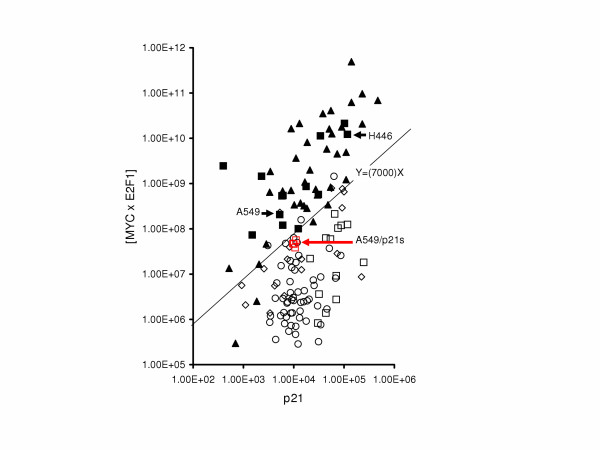
**Bivariate analysis of p21 and [MYC × E2F1]**. The X-axis represents p21 TA while the Y-axis represents the expression product of [MYC × E2F1]. The cut-off value is indicated by a line with the slope of 7,000. Open symbols represent non-malignant (normal) BEC samples, while filled-in symbols represent malignant BEC samples. All cultured BEC samples are indicated as squares. Primary normal BEC samples are indicated as circles, and primary normal parenchyma samples are indicated as diamonds. Primary malignant BEC samples are indicated as triangles. H446, A549 and A549/p21 clones are indicated.

Because the [MYC × E2F1]/p21 ITAI is a ratio between two values, the cut-off is best represented as a line on a bivariate plot. On a plot of all normal or malignant samples the ITAI threshold is represented as a line with a slope of 7,000 that passes through the origin (Figure 2). Thus, some malignant BEC have a very high p21 level and are above the cut-off only because they also have a very high [MYC × E2F1] value (e.g. H446), while others have a low p21 level and a relatively low [MYC × E2F1] level is sufficient to place them above the cut-off. In this way, the cut-off separates nearly all malignant from all normal samples. A549 is a good example of this phenomenon. The p21 TA level was higher than many normal BEC samples and the [MYC × E2F1] TA level was lower than some normal BEC samples. Yet, the p21 to [MYC × E2F1] ratio was above the ITAI cut-off.

### Expression of index genes and the ITAI in parental A549 and A549/p21 clones

**Figure 3:**
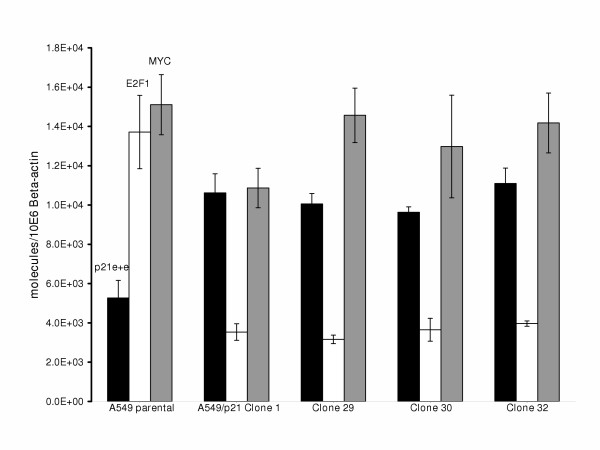
**Combined endogenous plus exogenous p21 expression and E2F1 and MYC expression for A549, A549/p21 clone 1, 29, 30, and 32**. Total p21 TA levels (e + e: endogenous plus exogenous), E2F1 TA levels, and MYC TA levels are indicated. Each column and error bar represents mean and standard deviation of at least triplicate measurements.

**Figure 4:**
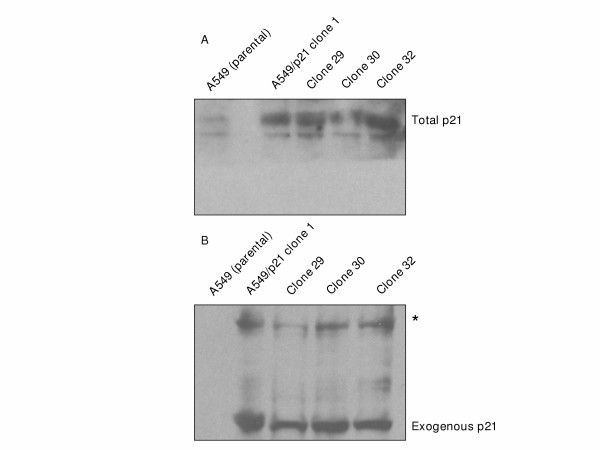
**Western blot analysis of combined exogenous and endogenous p21 protein and exogenous p21 protein expression from A549, A549/p21 clone 1, 29, 30, and 32 cell lysates**. **A) **Total p21 protein was analyzed in parental A549 or A549/p21 clones, which express stable low-level exogenous p21. Twenty μg of lysate were blotted on a PVDF membrane and incubated with anti-Waf1 (p21) (Ab-1) primary monoclonal antibody. **B) **Exogenous p21 protein was confirmed in the A549/p21 clones. Twenty μg of lysate were blotted on a PVDF membrane and incubated with anti-His Tag monoclonal antibody recognizing the Hexa-Histidine fusion tag of p21 transactivated from the expression vector contained in the stable A549/p21 clones. The asterisk indicates the non-specific antigen/antibody interaction.

Stable low-level expression of p21 RNA and protein were observed during screening of A549 clones 1, 29, 30, and 32, and these clones were used in all subsequent studies. Doxycycline did not increase expression of p21 in these lines. Total p21 TA value (combined exogenous p21 plus endogenous p21 expression-see Methods) was significantly (p < 0.05) increased two-fold in each of the A549/p21 clones compared to the parental A549 (Figure 3). The increase in total p21 at the RNA level was associated with a corresponding increase in total p21 protein measured by Western blot analysis (Figure 4a). StaRT-PCR confirmed the expression of exogenous p21 TA in the A549/p21 clones (data not shown), and exogenous p21 protein expression was confirmed by Western blot analysis of the Hexa-His-tagged p21 (Figure 4b).

**Figure 5:**
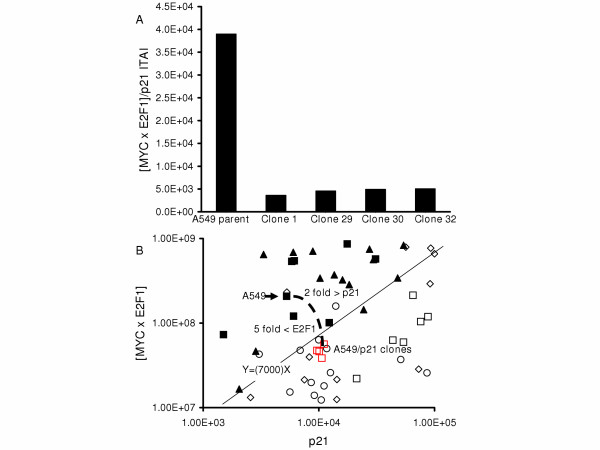
**ITAI values and bivariate analysis for A549, A549/p21 clone 1, 29, 30, and 32**. **A) **Mean TA value for each gene (from Figure 2) was used to calculate the ITAI. **B) **Bivariate analysis of p21 and [MYC × E2F1] for parental A549, A549/p21 clones and samples in their vicinity that demonstrates the 2-fold increase in p21, the 5-fold decrease in E2F1, and subsequent ITAI value that is below the cut-off threshold.

Expression of Hexa-His-tagged p21 was observed in each clone (lanes 2–5) but not in the parental A549 (lane 1). A higher molecular weight protein(s) was observed in the lysates from the clones suggesting a non-specific antigen/antibody interaction. This increase in total p21 expression was associated with a greater than 5-fold significant (p < 0.05) decrease in E2F1 TA in each clone (Figure 3). Further, the 2-fold increase in p21 TA and 5-fold decrease in E2F1 TA resulted in a greater than 10-fold significant (p < 0.05) decrease in the ITAI in each of the A549/p21 clones relative to parental A549 (Figure 5a), to levels below the cut-off threshold of 7,000 (Figure 2 and 5b). With the exception of A459/p21 clone 1, there was no significant statistical difference in MYC TA among the remaining stable transfectants and parental A549 (Figure 3). Further, among the A549/p21 clones, variation in expression of MYC, E2F1, and p21 and the resultant ITAI were insignificant.

### Growth kinetics of A549 and A549/p21 clones

**Figure 6:**
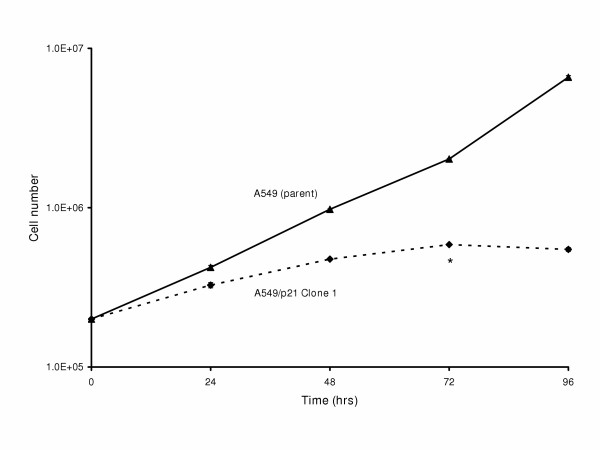
**Proliferation kinetics for A549, and A549/p21 clone 1**. Thirty mm Petri dishes were inoculated with 2 × 10^5 ^cells from either parental A549, clone 1, 29, 30, or 32, then incubated for 0, 24, 48, 72, or 96 hours. At each time point, cells were counted (total cell counts) and assessed for viability. Shown here are proliferation rates for parental A549 and A549/p21 clone 1 cell populations because while the growth rates are statistically significant between parental A549 and the A549/p21 clones, the proliferation rates among the clones were statistically insignificant. The experiment was repeated in triplicate and data reported as mean +/- standard deviation. The error bars are too small to visualize on this scale. The asterisk at 72 hrs indicates the time point when clonal populations were approximately 80% confluent and exhibited decreased viability.

Decreasing the ITAI to a level below 7,000 was associated with increased doubling time indicative of p21 effects on A549 proliferation kinetics. There was a nearly 2-fold increase in doubling time in each of the clones from 22.3 hrs for parental A549 to an average of 38.4 hrs in the four clones, in either antibiotic selection medium or antibiotic-free medium (Table 1). The rate of proliferation for the A549/p21 clones significantly (p < 0.05) decreased (0 hrs to 72 hrs) and dramatically decreased after 72 hrs with no significant change in number of cells between 72 and 96 hrs, and viability decreased at high saturation density (80% confluency) (Figure 6). Despite variable seeding densities (1 × 10^5^, 5 × 10^5^, and 1 × 10^6 ^cells) of the A549/p21 clonal populations, the cells ceased to proliferate and viability decreased at 80% confluency further suggesting p21-mediated contact inhibition at high saturation densities (data not shown). While the differences in proliferation rates were significant between parental A549 and the A549/p21 clones, there were no significant differences among any of the individual clones. Each of the A549/p21 clones was capable of approximately 15 total doubling times based on the estimated number of cells in the established initial foci (≈1 × 10^2 ^cells) compared to the estimated number of cells in the seventh passage, which was the last passage that they were capable of (data not shown). These passage limitations are similar to the passage limitations of cultured normal BEC [28].

**Table 1:** Doubling time for A549 and A549/p21 clones.

	**Doubling time (hours)^1^**	
**Sample**	**w/**	**w/o**
	**antibiotic^2^**	**antibiotic**
A549 (parental)	NA^3^	22.3
A549/p21 clone 1	39.3	38.5
clone 29	39.5	38.2
clone 30	39.3	39.3
clone 32	38.2	37.7

1) Doubling time was calculated by plotting the graph of cell count vs. time using the third degree polynomial. 2) Populations grown in 450 μg/mL G418 (minimum lethal dose 100). 3) No growth of parental A549 in medium containing selectable antibiotic; all populations grown in RPMI and 10% FBS.

### Flow cytometric DNA analysis for A549 and A549/p21 clones

**Figure 7:**
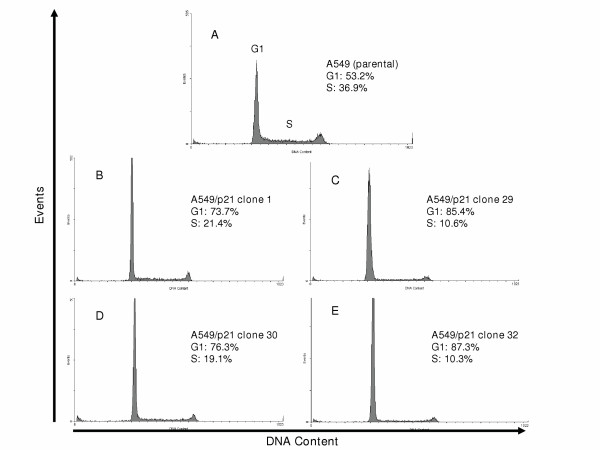
**Flow cytometric DNA analysis on A549, A549/p21 clone 1, 29, 30, and 32**. **A) **Determination of DNA content and events or cell number for parental A549 and A549/p21 clones constitutively expressing p21 **(B-E) **were performed as described in Methods and [50].

Decreasing the ITAI across the cut-off level between normal and malignant BEC was associated with p21-mediated cell-cycle arrest and/or delayed S-phase entry indicative of restored proliferation control. For the parental A549, 53.2% and 36.9% of cells were in G1 and S-phases respectively (Figure 7a). In contrast, in the A549/p21 clones, the G1 fractions were increased to an average of 80.7% (73.7%, 85.4%, 76.3%, and 87.3% in each respective clone) and the S-phase fractions were decreased to an average of 15.4% (21.4%, 10.6%, 19.1%, and 10.3% in each respective clone (Figure 7b–e)

## Discussion

Diagnostic tests, including those based on molecular diagnostic biomarkers, require establishment of a cut-off value to separate one clinically important phenotype from another. Cut-off values for multiple variables may then be combined through multivariate analysis to derive a diagnostic test with better accuracy than any of the individual cut-off values [5-7]. When attempting to approximate a complex phenotype through TA measurement, a significant limitation of multi-variate analysis is that it does not cause the individual variables to mathematically interact. It is not possible to deduce how the TA level of each gene scales with respect to a particular phenotype, nor how multiple genes interact at the TA level to give rise to a phenotype. It is likely that the scaling varies from one gene to another, and that interactions among the genes may involve both linear and non-linear functions. This is one reason that combination of TA values into ITAI leads to development of biomarkers with improved accuracy and ITAI biomarkers for clinical diagnostics have now been described [5-7]. It is important to recognize that the cut-off values for these ITAI represent lines on bivariate plots. One implication is that there is no prior reason that the best cut-off line will pass through the origin, as it does for the [MYC × E2F1]/p21.

A bivariate plot of p21 versus [MYC × E2F1] enables quantitative experimental investigation of the relationship between MYC, E2F1, and p21 individual transcript abundance values and the ITAI cut-off value that distinguishes normal from malignant BEC. For example, a malignant BEC line with a low [MYC × E2F1] value relative to most BC samples may be malignant if the p21 value also is relatively low. This was the case with parental A549. Although the A549 [MYC × E2F1] value is lower than in most malignant tissues (Figure 2), the ITAI value is above the cut-off threshold because the p21 level is low relative to the [MYC × E2F1] value. In contrast, a sample with a high p21 may be malignant if the [MYC × E2F1] value is sufficiently high, as was observed, for example, in the bronchogenic carcinoma cell line H446 (Figure 2, and [9]).

From these empirical observations, we selected the A549 cell line for subsequent experiments because its ITAI value was in close proximity to the cut-off threshold, and it was anticipated that even a relatively small increase in p21 TA value would be associated with restoration of cell proliferation control. Because it was anticipated that high constitutive expression of p21 would inhibit cell cycling to the extent that it would not be possible to isolate transfected clones, the goal was to obtain stable p21 transfectants that were under control of a tetracycline inducible promoter. Because the tetracycline repressor was not functional in the transfectants, exogenous p21 expression was not repressed in the absence of tetracycline, but was constitutively active. Thus, two selection processes were operative. On the one hand, there was antibiotic selection for clones with stable integration of the transfected p21 expression vector. On the other hand, survival and clonal expansion also was dependent on a p21 expression level low enough to allow cell cycling. Therefore, while expression and growth kinetics varied significantly between A549 and the stable, constitutively expressing p21 A549-derived clones, the observed lack of clonal variation (insignificant differences in expression and growth kinetics among the A549/p21 clones) supports these two selection processes. Each successfully isolated A549/p21 clone had total p21 expression that was elevated relative to parental A549, but was low enough to allow 15 doublings, which was sufficient for the studies reported here.

The absence of inducible clones could have been due to a number of factors, including promoter degradation, deleterious vector integration, and/or sub-optimal selection conditions affecting either the tetracycline repressor vector or the p21 expression vector.

In each A549/p21 clone we observed 1) a two-fold increase in p21, accompanied by 2) a 5-fold decrease in E2F1 TA and 3) that a 10-fold reduction in the ITAI was sufficient for each A549/p21 clone to cross the cut-off threshold of 7,000. This was associated with marked reduction in cell cycle progression and cell proliferation in the A549/p21 clones.

p21 exerts its inhibitory function in either late G1 or late G2 of the cell cycle [26,27]. Consistent with this, our results demonstrate that in the A549/p21 clones, cell cycle arrest and delayed progression through the cell cycle was mediated at late G1 based on the average increase in the fraction of cells in G1-phase and the average decrease in S-phase of the clones relative to the parental cell line. Moreover, because E2F1 expression is important for S-phase progression (reviewed in [29]) and is up-regulated in A549 due, in part, to p14ARF deletions, which normally serves to suppress E2F1 both directly [30] and through inhibition of MDM2 leading to activation of p53 [31], it is reasonable to conclude that down-regulation of E2F1 mediated by up-regulated p21 expression contributed to growth inhibition at late G1. Because RB1 (retinoblastoma) is intact in A549 and is a substrate of cyclin-cdk function, inhibition of these regulatory complexes and/or pRb directly [17] by p21 may have contributed to decreased or inhibited pRb phosphorylation. This, in turn, would be associated with diminished E2F1 dissociation, decreased E2F1 auto-up-regulation [32,33] and attenuated progression through the cell cycle. However, another possibility has been reported, where p21 has been shown to directly associate with E2F [34].

MYC TA level was not decreased considerably in the A549/p21 stable clones compared to the parental A549 in the context of a five-fold decrease in E2F1 TA level. This is in contrast to reports that in some contexts E2F1 is a transcription factor for MYC [35] and that reciprocal regulation occurs between MYC and p21 [36]. One possible explanation for this is that one or more transcription factors other than E2F1 contribute to MYC regulation. It is notable that a decrease in the ITAI to below the cut-off level, delay of cell cycle progression and re-establishment of proliferation control may be accomplished without significant alteration in MYC TA level, provided that sufficient alteration of p21 and E2F1 occur. Further, while MYC is a proven oncogene and increased expression of MYC causes oncogenic transformation in lung epithelial cells (reviewed in [37]), these studies indicate that regaining MYC regulation is sufficient, but not necessary to re-establish cell cycle control in malignant cells. Others have made observations in other models consistent with those described here. For example, Boxer et al. observed that mammary adenocarcinomas that showed down-regulation of MYC retained their malignant properties and that transformation of residual neoplastic cells was independent of MYC expression [38].

Normal BEC exhibit contact inhibition in culture at particular cell densities and cellular senescence (irreversible growth arrest) [28]. Cessation of proliferation and loss of viability in the A549/p21 clones after 72 hrs of continuous high saturation density likely was mediated by up-regulation of p21. This response has been observed in melanoma cells over-expressing p21 [39]. This p21 effect may be mediated in part through the up-regulation of H-cadherins [40] and/or T-cadherins independent of p53 [41]. Also, cell-to-cell contacts initiate prolonged nuclear localization of p21 that leads to growth arrest and apoptosis [39] and this effect may have been accentuated due to exogenous p21 up-regulation. Normal alveolar epithelial cells or A549 cells, exposed to sub-lethal doses of cigarette smoke, over-express p21 and undergo cellular senescence both *in vitro *and *in vivo *[42]. The cytokine, TGF-β, has been demonstrated to up-regulate p21 [43], and A549 cells treated with TGF-β over a long period were forced to undergo senescence and senescent A549 cells were not tumorigenic in nude mice [44]. Increased p21 expression has been associated with G1 growth arrest and senescence in hepatoma cells treated with ninjurin 1, a novel adhesion molecule that promotes regeneration in nervous tissue but senescence in liver tissue [45].

## Conclusion

In conclusion, characterization of the role of p21 in regulating cell proliferation and better quantification of the balance among cell proliferation control genes, as measured by TA values, was achieved through a moderate up-regulation of p21 in the A549 cell line. This allowed experimental quantitative confirmation that a cut-off level of 7,000 for the [MYC × E2F1]/p21 ITAI distinguishes between malignant and non-malignant phenotypes. This will guide development of more accurate diagnostic tests and more effective gene-targeted therapeutics.

## Methods

### Cell lines and culture conditions

The parental A549 cell line was cultured in RPMI 1640 with 10% fetal bovine serum (Clonetics, San Diego, CA). The A549/p21 clones 1, 29, 30, and 32 were continuously maintained in RPMI and 10% Tet-system approved FBS (Clontech, Palo Alto, CA) with 450 μg/mL Geneticin (G418 sulfate) (Invitrogen, Inc., Carlsbad, CA) unless otherwise noted.

Culture conditions for normal and malignant BEC were described previously [3]. Normal and malignant BEC proliferate optimally under different conditions [46]. The medium that is optimal for malignant BEC, RPMI with 10% fetal bovine serum (Clonetics, San Diego, CA), induces terminal squamous differentiation in normal BEC [28]. Conversely, malignant BEC do not divide in serum-free medium that is optimal for proliferation of normal BEC. Thus, the 13 malignant BEC lines were cultured in RPMI with 10% FBS and normal BEC from eight individuals (Clontech lot numbers: 10525, 17378, 6F0333, 6F0450, 17684, 17714, 6F0395, and 7F0075) were cultured in serum-free BEGM growth medium (Clonetics, San Diego, CA). In order to directly compare carcinoma cell lines to normal BEC under the same culture conditions, five of these normal BEC cell populations, 10525, 17378, 6F0333, 6F0450, and 17684 were incubated for 24 hours in RPMI with 10% FBS directly prior to assessment (Additional File 1).

### Primary tissue samples

Sixty-five primary normal BEC samples and thirty-one primary bronchogenic carcinoma samples (Additional File 1) were obtained under IRB approved protocols as described previously [6,8].

### Construction and cloning of p21 expression vector

The GeneStorm hORF Expression Vector (pcDNA3.1/GS), containing the complete cDNA sequence of the cyclin-dependent kinase inhibitor 1A (p21, Cip1) (Accession L25610) was obtained from Invitrogen, Inc., (Carlsbad, CA); The Gateway cloning system (Invitrogen, Inc., Carlsbad, CA) was used to create the p21 expression vector. Primers were designed to amplify the full-length p21 cDNA insert (Forward primer 5' end flanked by CACC, ^5'^CACCATGTCAGAACCGGCTGG^3' ^and reverse primer ^5'^TCAATGGTGATGGTGATGAT^3'^) were suitable for directional Topoisomerase (TOPO) cloning (Invitrogen, Inc., Carlsbad, CA) into pENTR/D-TOPO. To amplify the p21 complete cDNA from the pcDNA3.1/GS, the PCR conditions were 94°C/15 sec., 58°C/30 sec., and 72°C/60 sec. for 35 cycles (slope = 9.9) in an air thermocycler (Idaho Technology, Idaho Falls, Idaho). Each of five 10 μL PCR reaction mixtures contained 2 mM dNTPs, 10×, 30 mM MgCl_2_, template (pcDNA3.1/GSp21), forward and reverse primers (0.1 μg/μL), Platinum Pfx DNA Polymerase (Invitrogen, Inc., Carlsbad, CA), and RNase-free water. After amplification, the five reactions were combined and electrophoresed in a 2% (NuSieve/SeaKem) agarose gel containing ethidium bromide. The PCR product was visualized under UV and extracted following the Qiagen gel extraction protocol (Qiagen, Santa Clarita, CA). The TOPO reaction was performed according to manufacturer's instructions. The pENTR/D-TOPO-p21 vector was isolated, quantified, and sequenced to confirm integrity and orientation. Next, the Gateway™ (Invitrogen, Inc., Carlsbad, CA) LR site-specific recombination system was used according to manufacturer's instructions to recombine the p21 ORF from the entry vector (pENTR/D-TOPO-p21) with the destination vector (pT-REx-DEST30), which contains two tetracycline operon (TetO2) sites within the human CMV promoter for tetracycline-regulated expression of the inserted gene.

### Stable transfection of cloned vectors

A549 cells were trypsin-dissociated, and each well of a 24-well dish was inoculated with 10^5 ^cells (90–95% confluent on the day of transfection) in 500 μL of RPMI and 10% Tet-system approved FBS (Clontech, Palo Alto, CA) without antibiotics. Twenty-four hours later, A549 cells were transfected with the pcDNA/TR vector (Invitrogen, Inc., Carlsbad, CA) that expresses the Tet-repressor. For each well of cells transfected, 1 μg of vector DNA (pcDNA/TR) was diluted into 50 μL of Opti-MEM I Reduced Serum Medium (Gibco, Carlsbad, CA). Two microliters of Lipofectamine 2000 (LF2000) (Invitrogen, Inc., Carlsbad, CA) was diluted into 50 μL of Opti-MEM I Medium (Gibco, Carlsbad, CA) and subsequent transfection was performed according to LF2000 manufacturer's instructions. At 24 hours post-transfection, the cells from each well were trypsin-dissociated and inoculated into corresponding 10 cm plates containing fresh growth medium, RPMI and 10% Tet-system approved FBS (Clontech, Palo Alto, CA) without antibiotics. The next day, selective medium was added containing blasticidin S-HCl (Invitrogen, Inc., Carlsbad, CA) at the A549 minimum 100% lethal dose (LD_100_) of 7 μg/mL. Within one week, individual foci developed, and each of these were individually dissociated, transferred to a single well in a 24-well dish and cultured in selection medium. The pcDNA/TR-A549 clonal populations were expanded and then sequentially transfected with the pT-REx-DEST30-p21 response vector by means described above but using the A549 minimal LD_100 _for Geneticin (G418 sulfate) (Invitrogen, Inc., Carlsbad, CA) at 450 μg/mL as the selection antibiotic pressure. The pcDNA/TR/p21-A549 transfectants were maintained under dual blasticidin S-HCl and Geneticin selection. Lowest basal and the highest tetracycline-induced p21 TA level after addition of 1 μg/mL of doxycycline was assessed in passage 4 cells from each clone, with passage 1 being the passage of the initial double antibiotic resistant clones.

The initial screen for inducible exogenous p21 expression was done on cells from passage 4 of the A549/p21 clones. All mRNA, protein, flow cytometric, and growth kinetic assessments were performed at passage 5 and compared to parental A549 with an equivalent number of passages post-transfection.

### RNA extraction and reverse transcription

For cultured or primary cells, pelleted cells were dissolved in TriReagent (Molecular Research Center Inc., Cincinnati, OH) and total RNA was extracted according to manufacturer's instructions and previously reported methods [47]. Then, approximately 1 μg of total RNA was reverse transcribed using oligo-dT primer and M-MLV reverse transcriptase (Invitrogen, Inc., Carlsbad, CA) as described previously [48].

### Quantitative standardized RT (StaRT)-PCR

StaRT-PCR was used for all TA measurements [2-4]. For TA measurement of each gene, cDNA from a cell or tissue sample of interest was mixed with the internal standard (IS) for the gene within a standardized mixture of internal standards (SMIS). The presence of SMIS in each reaction enabled end-point quantification and controlled for all known sources of variation in PCR amplification, including inter-sample variation in the presence of gene-specific inhibitors. After PCR, 1 μL of the PCR product was electrophoresed and quantified using an Agilent 2100 bioanalyzer according to manufacturer's recommended protocol (Agilent Technologies, Palo Alto, CA), and then each gene was quantified based on the ratio of the endogenous gene product (NT, native template) to its respective internal standard (IS) within the SMIS [2-4,49]. Because the initial concentration of IS added into the PCR reaction was known and it amplified with the same kinetics as the NT, the initial NT concentration could be determined through a ratio with the IS. Second, the calculated number of target gene NT molecules was divided by the calculated number of β-actin NT molecules to control for loading differences. Although β-actin was used as the reference gene for all measurements, it was possible to recalculate the data relative to any other single gene or combination of genes that was measured. Primers-(obtained from Gene Express, Inc; GEI, Toledo, OH) for TA measurement in all cultured and primary samples of each gene comprising the ITAI (MYC, E2F1, and p21) and β-actin were described previously [2]. The p21 primers hybridize in the 3' untranslated region of the p21 cDNA. Therefore, in order to measure the p21 TA in A549 and the stable A549/p21 transfectants, another set of p21 primers (not commercially available) were designed to discern between exogenous expression of p21 (transcribed from the CMV-regulated p21 expression vector) and total p21 expression (endogenous plus exogenous). To specifically measure exogenous p21 TA, the reverse primer was anchored in the 3' Hexa-His-tag sequence that flanks the p21 ORF (reverse NT sequence, ^5'^GTGATGGTGATGATGACCG^3' ^while the forward was ^5'^ACCCTTGTGCCTCGCTCAG^3'^). In order to measure total p21 exogenous plus endogenous TA level, forward and reverse primers ^5'^GCCTGCCCAAGCTCTACCT^3' ^and ^5'^GAGAAGATCAGCCGGCGTT^3'^, respectively, were designed that hybridize in the coding region of p21 and not the 3' Hexa-His-tag sequence. Previously reported data for TA measurement of MYC, E2F1, and p21 in cultured normal BEC, all malignant BEC except H146 (Additional File 1), and primary BEC were obtained using SMIS prepared in this laboratory [2]. More recent data were obtained using the same methods except SMIS used were those commercially available for StaRT-PCR from Gene Express, Inc. (Toledo, OH). The previously published data then were converted to values based on the commercial SMIS. The conversion factor for each gene between the two SMIS was determined by evaluating a set of cDNA samples with each SMIS.

### Western blotting and antibodies

Parental A549 and A549/p21 cells were lysed by three consecutive freeze-thaws in a 0.25 M Tris lysis buffer (Invitrogen, Inc., Carlsbad, CA) and total protein concentration was determined colorimetrically by the bicinchoninic acid (BCA) assay (Pierce, Inc., Rockford, IL). Samples were solubilized in NuPAGE LDS Sample buffer (Invitrogen, Inc., Carlsbad, CA) and 20 μg was loaded on a denaturing 7% NuPAGE Tris-Acetate gel (Invitrogen, Inc., Carlsbad, CA). Protein bands were transferred to Invitrolon PVDF membranes (Invitrogen, Inc., Carlsbad, CA) as described by the manufacturer. Then, the membranes were blocked with 5% non-fat dry milk in 1× TBST (blocking buffer) for 1 hr at room temperature. For determination of exogenous p21 protein expression, blots were incubated overnight with Anti-His Tag Mab (Novagen, Madison, WI) (1:2000) recognizing the Hexa-Histidine fusion tag of p21 transactivated from the expression vector contained in the stable A549/p21 clones. Blots were then washed with 1× TBST and incubated with the secondary antibody, HRP-conjugated anti-mouse IgG (Fisher, Pittsburgh, PA) (1:5000) for 1 hr at 37°C. Blots were washed three times with 1× TBST and once with 1× TBS. Detection was performed by the commercially available ECL detection system (Santa Cruz biotechnology, Santa Cruz, CA). For determination of total p21 protein expression (exogenous plus endogenous), blots were incubated with Anti-Waf1 (Ab-1) Mab (Oncogene Science, Cambridge, MA) (1:500). HRP-conjugated goat anti-mouse IgG (Santa Cruz biotechnology, Santa Cruz, CA) (1:2000) was used as the secondary antibody. Methodology was the same as described above. The Hexa-Histidine tag that flanks the ORF of p21 was necessary for determining expression of p21, both mRNA and protein, that was exogenously synthesized. To date, there is no evidence suggesting deleterious effects of Hexa-Histidine tags on the function of p21. Further, because the relative expression of His-tagged p21 was low in the A549/p21 clones, unexpected alterations in expression profiles and phenotype are less likely.

### Cell proliferation characteristics

Sub-cultures of the clonal populations were maintained and periodically sub-cultured for determination of maximum number of passages at optimal confluency (50% or 5 × 10^6 ^cells/T75 flask). To calculate the number of population doublings at each passage, the log2 of the number of cells inoculated was subtracted from the log2 of the cell count for the cells harvested. This information was used to determine the number of population doublings for each clone.

Cell doubling time was determined by counting cells from trypsinized monolayers. Viability was assessed based on trypan-blue (Sigma-Aldrich, Saint Louis, MO) exclusion. 30 mm Petri dishes containing 2 × 10^5 ^cells from either parental A549, clone 1, 29, 30, or 32 were used for each time point (0, 24, 48, 72, and 96 hrs). The stable clones were maintained with RPMI and 10% FBS containing 450 μg/mL G418. In order to rule out potential effects of the antibiotic on cell proliferation, the experiment was repeated in antibiotic-free medium. Both experiments were repeated in triplicate and the doubling time was calculated by plotting the graph of cell count versus time using the third degree polynomial.

For further demonstration of growth characteristics of the A549/p21 clonal populations, variable numbers of cells (1 × 10^5^, 5 × 10^5^, and 1 × 10^6 ^cells) were inoculated per T75 flask for determination of percent confluency at which cells ceased dividing.

### Flow cytometry and determination of DNA content

To determine DNA content and cell cycle phase, cells from the parental A549 and clones 1, 29, 30, and 32  were harvested at 50% confluency. Next, 1 × 10^4 ^cells were prepared for analysis by the propidium iodide, detergent, and trypsin nuclei method as described by Vindelov, et al., 1982 [50]. The cells were then evaluated for DNA content and cell number on an EPICS Elite Flow Cytometer (Beckman-Coulter Corp., Miami, FL, USA). Values were automatically calculated by Multicycle AV software (Phoenix Flow Systems, Inc., San Diego, CA, USA). Aggregates were excluded by pulse height versus pulse area.

### Graphic and statistical analysis

Sensitivity and specificity were determined by 2 × 2 table analysis, and numerical variable differences were determined by paired-sample T-tests (p-value less than 0.05 was considered statistically significant) using SPSS 11.5.1 for Windows (SPSS, Chicago, IL). Determination of mean TA and cell number, standard deviation of TA and cell number, cell doubling time, and creation of graphs were accomplished using Excel 2000 (Microsoft Corp., Redmond, WA).

## Competing interests

ELC and JCW each have significant equity interest in Gene Express, Inc., which produces and markets StaRT-PCR reagents used in these studies.

## Authors' contributions

TGG conceived and designed experiments, and was responsible for TA measurement in cultured samples, construction and cloning of p21 expression vector, stable transfection of vector and construction of A549/p21 clones, preparation of the exogenous and endogenous and exogenous p21 SMIS, TA measurement in A549 and A549/p21 clones, Western blot analysis, cell proliferation characteristic analyses, prepared cells for flow cytometric DNA analysis, statistical analysis, and was the primary author of this manuscript. MWH was responsible for TA measurement in cultured samples and critical review of the manuscript. ELC was responsible for TA measurement in primary samples and critical review of the manuscript. JCW coordinated and obtained funding for the study and revised the manuscript.

## Supplementary Material

Additional File 1 **TA, ITAI values and descriptions of all samples** Click here for file
